# The Association of Tau With Mitochondrial Dysfunction in Alzheimer's Disease

**DOI:** 10.3389/fnins.2018.00163

**Published:** 2018-03-22

**Authors:** Ying Cheng, Feng Bai

**Affiliations:** ^1^Department of Neurology, Affiliated ZhongDa Hospital, School of Medicine, Southeast University, Nanjing, China; ^2^Department of Neurology, Drum Tower Hospital, Medical School of Nanjing University, Nanjing, China

**Keywords:** Alzheimer's disease, tau, mitochondrial transport, mitochondrial dynamics, mitochondrial dysfunction

## Abstract

Increasing evidence suggests that abnormally hyperphosphorylated tau plays a vital role in the pathogenesis of Alzheimer's disease (AD). Mitochondrial dysfunction also has a recognized role in the pathophysiology of AD. In recent years, mitochondrial dysfunction has been strongly associated with tau pathology in AD. Overexpression of hyperphosphorylated and aggregated tau appears to damage the axonal transport, leading to abnormal mitochondrial distribution. In addition, pathological tau impairs mitochondrial dynamics by regulating mitochondrial fission/fusion proteins, and further causes mitochondrial dysfunction and neuronal damage. Moreover, mitochondrial dysfunction is also involved in promoting tau pathology in AD. In this article, we evaluate the relationship between phosphorylated tau and mitochondrial dysfunction in AD.

## Introduction

To determine the physiopathologic mechanism of AD for effective prevention and treatment, several modeling hypotheses have emerged, although the exact cause of AD is still unclear. Hardy and Higgins (1992) first formulated the amyloid hypothesis in 1992, which was widely accepted at the time but is much questioned now (Hardy and Selkoe, 2002). In the amyloid hypothesis, amyloid precursor protein (APP) is cleaved by β and γ-secretases and produces amyloid beta 42 (Aβ42) fragments. Aβ42 fragments aggregate into insoluble extracellular fibrils of neuritic plaques (NPs), and neurofibrillary tangles (NFTs) are subsequently formed. However, several molecular and genetic abnormalities and inconsistent clinical correlations identified in some research cannot be explained by this hypothesis. Furthermore, no new drug based on the amyloid hypothesis has been approved over the past decade. Recently, another phase III clinical trial of Alzheimer's drug targeting Aβ was shuttered because the drug has no chance of benefiting AD patients. Given the failures of the clinical trials based on the amyloid hypothesis, the tau hypothesis, an alternative pathway, is now attracting increasing attention. It is well-known that NFTs, which are composed primarily of hyperphosphorylated tau protein (Grundke-Iqbal et al., 1986; Nukina and Ihara, 1986), have been proposed as the second pathological hallmark of AD. In addition, increasing evidence suggests that abnormally hyperphosphorylated tau plays a vital role in the pathogenesis of AD. Hyperphosphorylated tau can lead to microtubule dysfunction, impair axonal transport of organelles including mitochondria, and result in synaptic dysfunction (Ittner and Götz, 2011; Cai and Tammineni, 2017). Mitochondrial dysfunction has been indicated as an underlying mechanism of AD pathophysiology. Accumulating evidence indicates a strong association of tau pathology with mitochondrial dysfunction in AD (Eckert et al., 2014). Overexpression and hyperphosphorylation of tau appear to impair mitochondrial axonal transportation, mitochondrial dynamics and function, and finally neuronal health (Eckert et al., 2014). In this article, the link between phosphorylated tau and mitochondrial dysfunction in AD is evaluated.

## Tau protein

Tau as a major microtubule-associated protein plays a significant role in neuronal processes. In adult human brains, high heterogeneity of the tau protein is apparent; there are six different tau isoforms, all of which are derived from a single gene by alternative mRNA splicing. The six isoforms of tau protein differ from each other in the number of microtubule-binding domains (3R/4R) (Lee et al., 1989) and in the presence or absence of either one or two projection domains (0N/1N/2N). Between the microtubule-binding domain and projection domain lies a basic proline-rich region (155–242), which contains abundant phosphorylation sites (Binder et al., 1985). The six isoforms appear to be broadly functionally similar, but each is likely to have precise, and to some extent distinctive, physiological roles. These isoforms appear to be differentially expressed during development. Normally, the 3R and 4R tau isoforms are expressed in a one-to-one ratio in most regions of adult brains (Ballatore et al., 2007).

The interaction of the proline-rich region of tau with the microtubule-surface contributes to microtubule stabilization (Amos, 2004). Phosphorylation as the most prominent post-translational modification of tau plays an important role in the dynamic equilibrium of tau with the microtubules (Arnold et al., 1996; Liu et al., 2004; Mazanetz and Fischer, 2007). It is well established that the serine/threonine-directed phosphorylation of tau directly regulates the binding affinity of tau for microtubules (Mazanetz and Fischer, 2007). The nonequilibrium of tau binding to the microtubules results in aggregation and fibrillization of tau and dysfunction of microtubules (Kuret et al., 2005). The microtubule network plays an essential role in axonal transport. It is likely that the resultant dysfunction of microtubules further leads to abnormal axonal transport and synaptic dysfunction. By modulating the microtubule network, tau has profound effects on axonal transport, which allows signaling molecules, trophic factors, and essential organelles including mitochondria and so on, to travel along the axons. Thus, tau contributes to vital structural and regulatory cellular functions.

## Tau impairs mitochondrial transport

To meet high energy demands and regulate calcium buffering of neuronal cells, efficient delivery of mitochondria in neurons is essential. The delivery of mitochondria is the task of microtubules, which perform a “rail track” function. Mitochondria are cargoes that are delivered by microtubule-associated proteins, including tau, across axon into synapses (Wang et al., 2015). Observations from studies of different cellular and mice models of AD show that overexpression and hyperphosphorylation of tau impair localization and distribution of mitochondria (Ebneth et al., 1998; Kopeikina et al., 2011; Shahpasand et al., 2012; Rodríguez-Martín et al., 2013), which further cause defects in axonal function and loss in synapses (Cabezas-Opazo et al., 2015; Wang et al., 2015).

The distribution of mitochondria in neurites containing tau aggregates was disrupted in an age-dependent manner in the rTg4510 mouse model (Kopeikina et al., 2011). Similar alteration of mitochondrial localization observed in human AD brains further confirmed the association of tau accumulation with mitochondrial translocation deficits (Kopeikina et al., 2011). Additionally, the reduction of soluble tau improved the aberrant mitochondrial trafficking in the rTg4510 mouse model (Kopeikina et al., 2011). In an axonal study of the squid AD model, it was filamentous rather than soluble forms of hyperphosphorylated tau that inhibited anterograde fast axonal transport through activating glycogen synthase kinase 3 (GSK3) and axonal protein phosphatase 1 (PP1) (Kanaan et al., 2011). Increased expression of glycogen synthase kinase-3β (GSK-3β) and the p25 activator of cyclin dependent kinase 5 were found to pause the mitochondrial movement in cortical neurons (Morel et al., 2010). At the same time, inhibition of GSK-3β reversed axonal transport disrupted by overexpression of tau in *Drosophila* (Mudher et al., 2004). These results show that mitochondrial transport is influenced by overexpression of tau, especially the aggregated tau. It is indicated that GSK-3β is involved in regulating overexpressed tau-induced mitochondrial translocation.

Abnormal phosphorylation of tau at AT8 sites (Ser199, Ser202, and Thr205) inhibited mitochondrial movement and affected mitochondrial distribution along the axons of cortical neurons in the mouse brain, which may contribute to the axonal degeneration (Shahpasand et al., 2012). As a possible underlying mechanism, it was reported that in K369I mutant tau transgenic K3 mice, phosphorylated tau trapped kinesin motor protein complex JIP1 in the soma. This aberrant JIP1 translocation caused cargo-selective (such as mitochondria) impairment of axonal transport (Ittner et al., 2009). Observation of pathological interaction of tau with JIP1 and trapping of JIP1 in the soma in AD patients further supports these findings in the mouse model (Ittner et al., 2009), while loss of axonal mitochondria enhances the abnormal phosphorylation and the toxicity of tau. Reduction of axonal mitochondria—caused by RNAi-mediated knockdown of Miro, an adaptor protein involved in axonal transport of mitochondria—led to abnormal tau phosphorylation at AD-related phosphorylation site Ser262 (Iijima-ando et al., 2012). These studies show a perishing interplay between abnormal phosphorylated tau and impaired mitochondrial distribution. A possible signaling pathway underneath the interplay is implicated. By trapping kinesin motor protein complex JIP1, hyperphosphorylated tau disrupts the transport of mitochondria into the axons and synapses. Loss of axonal mitochondria fails to maintain dynamic equilibrium of tau with the microtubules in the axons and enhances the abnormal phosphorylation of tau, which is further entangled in a vicious circle.

In a study with various neuronal and nonneuronal cell lines, it is shown that tau impaired not only axonal transport of organelles (including mitochondria), but also the transport of amyloid precursor protein (APP) (Mandelkow et al., 2003). Retarded APP transport caused by tau indicates a possible relationship between two vital pathogenic factors in AD. In primary neurons of a tau knockout mouse model, tau was required in the Aβ-induced impairment of axonal transport, and the inhibition of tau overexpression protected against the Aβ-induced defective axonal transport of mitochondria and neurotrophin receptor TrkA (Vossel et al., 2011). Evidence from these studies suggests a predominant role of tau in the mitochondrial transport impairment in AD.

Various pathological forms of tau, such as hyperphosphorylated and aggregated forms, have been indicated as noxious (Kopeikina et al., 2012). Current studies show that overexpression of hyperphosphorylated and aggregated forms of tau plays a vital role in the impairment of axonal transport, and also that the resultant aberrant localization and distribution of mitochondria further cause axonal damage and synapse degeneration.

## Tau impairs mitochondrial dynamics

Mitochondria are dynamic organelles that constantly undergo fission and fusion activities. Balanced mitochondrial fission and fusion are beneficial processes (Westermann, 2012). Firstly, mitochondrial fission/fusion dynamics promote mitochondrial distribution along axons into synapses (Chen et al., 2007). Secondly, they intermix metabolites and mitochondria to enable the establishment of mitochondrial networks, which are essential for transmitting mitochondrial membrane potential (Skulachev, 2001) and for buffering calcium signals (Szabadkai et al., 2006). Also, they can separate defective mitochondrial constituents to defend against reactive oxygen species (ROS) damage during aging (Westermann, 2008). In brief, balanced mitochondrial fission/fusion dynamics are essential to meet high energy demands and facilitate neuroprotective effects (Chen and Chan, 2009; Santos et al., 2010). A group of guanosine triphosphatases (GTPases) has been found to regulate mitochondrial fission and fusion processes. Dynamin-like protein 1 (DLP-1 or Drp1) and a small molecule fission protein-1(Fis1) are involved in the regulation of the fission process (Chan, 2006; Su et al., 2010b). The fusion process is regulated by mitofusin 1 (Mfn1), mitofusin 2 (Mfn2) and optic atrophy protein 1 (OPA-1) (Cipolat et al., 2004; Youle and van der Bliek, 2012). Mitochondrial dynamics within the neuronal environment are mediated by the above-listed proteins. Mitochondrial morphology and function is tightly regulated in response to cellular and environmental stresses (Picard et al., 2013). It is reported that mitochondrial dysfunction and abnormal morphology are prominent features of AD (Wang et al., 2014b; Gao et al., 2017; Guo et al., 2017). It is generally accepted that the dynamic balance of mitochondrial fission and fusion is disturbed in AD, shifting toward immoderate fission (Wang et al., 2009; Bonda et al., 2010).

Abnormal interaction between hyperphosphorylated tau and Drp1 caused an excessive mitochondrial fission process and led to the degeneration of mitochondria and synapses in brain tissues of APP, APP/PS1, and 3xTg-AD mice. Similar results found in brain tissues from AD patients further improve the validity of the role of hyperphosphorylated tau in impaired mitochondrial dynamics (Manczak and Reddy, 2012a). In addition, reduction of Drp1 was able to protect against hyperphosphorylated tau-induced mitochondrial and synaptic impairment (Kandimalla et al., 2016). In a recent study about the effects of different forms of tau on mitochondrial dynamics, it is found that truncated tau fragmented mitochondria combined with reduced levels of OPA-1 (Pérez et al., 2017). In contrast, human wild-type full length tau (htau) impaired the balance of mitochondrial dynamics through increasing fusion proteins OPA-1, Mfn1, and Mfn2 and enhancing mitochondrial fusion activity in the HEK293 cell line and primary hippocampal neurons of rats (Li et al., 2016). Previous studies suggest that mitochondrial fusion is a protective process, while an excessive fission process is a sign of malfunction. Adverse results of these two studies may be due to the difference among the sensibility of diverse cell lines responding to the toxicity of tau. In Li et al.'s study, the htau promoted mitochondrial fusion and increased the cell viability at 48 h. After overexpression of htau, axonal degeneration and neurite loss were observed (Li et al., 2016). It is proposed that accumulated tau ameliorates acute apoptosis by promoting mitochondrial fusion at an early stage while it causes neuronal degeneration with increasing mitochondrial fission at later stage (Li et al., 2007; Wang et al., 2010, 2014a).

The dynamic balance of mitochondrial fission/fusion and normal mitochondrial distribution are essential for maintaining mitochondrial function (Chen and Chan, 2009). Evidence from these studies suggests that pathological forms of tau play a significant role in the impairment of mitochondrial fission/fusion dynamics in AD, mainly through a molecular mechanism of increasing mitochondrial fission protein such as Drp1 and decreasing fusion protein including OPA-1, Mfn1/2. Tau-induced abnormal mitochondrial dynamics and impaired mitochondrial distribution may further lead to mitochondrial dysfunction.

## Tau pathology and mitochondrial dysfunction in AD

Mitochondrial dysfunction plays a fundamental role in the pathogenesis of AD (Moreira et al., 2010). Defects in mitochondrial function are manifested by a variety of indicators, including decreased ATP synthesis, increased ROS production, impaired oxidative phosphorylation system (OXPHOS) complexes and antioxidant enzymes (Cabezas-Opazo et al., 2015). It is proposed that pathological forms of tau impair mitochondrial function. In both cell culture and transgenic mice studies, overexpression of tau inhibits mitochondrial function by decreasing the activity of complexes and antioxidant enzymes and repairing ATP synthesis and synaptic function (Eckert et al., 2011; Wang et al., 2015; Li et al., 2016). Perinuclear distribution of mitochondria causes ATP depletion, oxidative stress and even synaptic dysfunction (Chen and Zhong, 2014; Wang et al., 2014b). It is also found that N-terminal-truncated tau localized in mitochondrial membrane impaired ATP synthesis and membrane potential (Atlante et al., 2008). It is shown that overexpression and mislocalization of tau impair mitochondrial function mainly through decreasing ATP production and increasing oxidative stress, finally leading to synaptic dysfunction in AD.

Interestingly, indicators of mitochondrial dysfunction found in the AD patients are also evidenced in different AD-related mice models expressing pathological tau. Abnormal interaction of phosphorylated tau with voltage-dependent anion channel 1 protein (VDAC1) observed in AD patients was also found in APP, APP/PS1, and 3xTg-AD mice models (Manczak and Reddy, 2012b). It broke the balance of the opening and closure of mitochondrial pores and led to mitochondrial dysfunction (Manczak and Reddy, 2012b). The P301L tau transgenic mice presented notable mitochondrial dysfunction. Brains of these mice showed that mitochondrial complexes activities were significantly reduced, especially complex I and V. Moreover, impaired ATP synthesis together with decreased mitochondrial respiration and increased ROS levels were also noticed (David et al., 2005). In the triple transgenic AD mouse model that generated from cross-breeding of P301L tau transgenic pR5 mice and APP_swe_PS2_N141I_ double-transgenic APP152 mice, compromised mitochondrial function was notably observed (Rhein et al., 2009). Proteomic and functional studies found that expressions and activities of mitochondrial complex I and IV were significantly deregulated, and deregulation of complex I was remarkably tau-dependent. Additionally, these mice also exhibited evident lacking of ATP synthesis, increased ROS production and depolarized mitochondrial membrane potential (Rhein et al., 2009). In another triple transgenic AD mice (3xTg-AD) with transgenes APP_swe_, PS1_M146V_, and Tau_P301L_, mitochondrial impairment was early evidenced by impaired mitochondrial respiration, and decreased pyruvate dehydrogenase (PDH), as well as increased oxidative stress (Yao et al., 2009).

## Discussion

We have discussed here the effects of tau overexpression on mitochondrial transport, dynamics and function in the pathogenesis of AD. It is demonstrated that pathological forms of tau impair mitochondrial function through three aspects including mitochondrial transport, dynamics and bioenergetics. In spite of that, some AD mice models exhibited cognitive impairment before observation of NFTs. This evidence raises doubts about whether pathological tau is a trigger of the mitochondrial abnormalities that are one of the earliest features in AD. It is postulated that mitochondrial impairment may also modulate the pathological hallmark tau in AD (Mondragón-Rodríguez et al., 2013). However, the underlying mechanism between mitochondrial dysfunction and tau pathology in AD remains elusive.

Mitochondria are the main source of ROS, and mitochondrial oxidative damage is significantly associated with AD (Tönnies and Trushina, 2017). In a previous study, chronic oxidative stress in cultured neural cells induced by inhibition of glutathione synthesis, elevated phosphorylation of tau at AD-specific phospho-sites. It suggests that chronic oxidative stress is able to increase phosphorylation of tau (Su et al., 2010a). Treating cultured neuronal cells with ROS mimicking mitochondrial oxidative stress, promotes tau phosphorylation (Ibáñez-Salazar et al., 2017) by increasing the activity of GSK-3β (Lovell et al., 2004). In addition, astrocytes-mediated fatty acid oxidative products induced tau hyperphosphorylation in neurons; further co-treatment of these neurons with an antioxidant decreased levels of tau hyperphosphorylation. This study provides a direct link between oxidative stress and AD-relevant tauopathy (Patil and Chan, 2005). Treating mice lacking superoxide dismutase 2 (SOD2) with catalytic antioxidant could decrease the levels of AD-associated tau hyperphosphorylation, which indicates that mitochondrial oxidative stress contributes to abnormal hyperphosphorylation of tau (Melov et al., 2007). In addition, mitochondrial complex I inhibitor annonacin mediated redistribution of tau from the axons to the cell soma in cultured neurons (Escobar-Khondiker et al., 2007). In summary, accumulated mitochondrial dysfunction contributes to the progressive development of AD, possibly through a mechanism whereby mitochondrial oxidative stress promotes abnormal phosphorylation of tau. However, more studies are needed to find supportive evidence.

Mitochondria dysfunction, as a prominent early feature in the pathogenesis of AD, leads to phosphorylation and aggregation of tau. Meanwhile, pathological tau impairs the mitochondrial axonal transportation, mitochondrial dynamics and function. Accumulated mitochondrial impairment and tau pathology in a vicious circle affect neuronal and synaptic function, leading to memory loss and cognitive impairment in AD. The relationship between pathological tau and mitochondrial dysfunction in AD is like the story of the chicken and the egg. More evidence is needed to decide whether mitochondrial dysfunction is the cause or consequence of pathological tau. Moreover, further experiments focusing on the link between mitochondrial dysfunction and pathological tau may be helpful for developing therapeutic targets.

## Author contributions

FB has been responsible for providing ideas and polishing the manuscript. YC takes charge of searching for evidence and writing the manuscript.

### Conflict of interest statement

The authors declare that the research was conducted in the absence of any commercial or financial relationships that could be construed as a potential conflict of interest.
